# Age and Transmissible Spongiform Encephalopathies

**DOI:** 10.3201/eid1006.031130

**Published:** 2004-06

**Authors:** Dennis M. Heisey, Damien O. Joly

**Affiliations:** * United States Geological Survey, Madison, Wisconsin, USA;; † University of Wisconsin, Madison, Wisconsin, USA

**Keywords:** age, follicular dendritic cells, prion

**To the Editor:** Bacchetti (*1*) notes "Our findings suggest that the possibility should not be discounted that biological factors peaking in the third decade of life may promote variant Creutzfeldt-Jakob disease (vCJD) prion replication and consequent development of disease." Such age specificity of disease risk may be a general feature of transmissible spongiform encephalopathies, which suggests that a general mechanism should be sought. A likely candidate for this mechanism is senescence-related immune system defects.

In a study of scrapie outbreaks in four sheep flocks, the incidence of clinical cases peaked in sheep 2–3 years of age, despite very different forces of infection at work and very large differences in disease incidence (*2*). Similar age specificity has been observed in cattle infected with bovine spongiform encephalopathy (*3*), which is believed to be the causal agent of variant Creutzfeldt-Jakob disease. There is evidence that an age-specific peak in prevalence also occurs in chronic wasting disease, a laterally transmitted spongiform encephalopathy of North American cervids, specifically elk, mule deer, and white-tailed deer. For example, data on prevalence of chronic wasting disease in mule deer (Figures 4B and 4A of [4]) suggest the existence of age-specific peaks. In aggregate, these observations suggest that a general mechanism might produce the marked decline in disease risk as age increases.

In 1979, Dickinson and Outram (*5*) conjectured that, in some experiments, scrapie responsiveness is the opposite of what one normally expects with an infection, "raising the possibility that, far from being inimical, some part of the host's immune system is essential and may even play the role of a Trojan Horse for these agents when infection occurs by a peripheral route." This theory appears well founded for transmissible spongiform encephalopathies in general. Disease-associated forms of resistant prion protein (PrP^Res^) are likely transported from the gut to lymphoid tissue by cells such as migrating intestinal dendritic cells (*6*). Once in the lymphoid tissue PrP^Res^ appears to be amplified by follicular dendritic cells (*6*) and then enters the nervous system. Defects in either the complement pathway or follicular dendritic cells result in resistance to peripheral scrapie infection (*7**,**8*), and this resistance likely occurs for peripheral transmissible spongiform encephalopathy infections in general.

Both in vitro and in vivo animal and human studies demonstrate age-related declines in both humeral and cellular components of the immune system (*9*). In old (23 months) mice, the normal functioning of follicular dendritic cells appears to be strongly impaired when compared with young mice (*10*); according to researchers, "Antigen transport was defective and only a small fraction of antigen transport sites developed." (*10*). Furthermore, follicular dendritic cells were ultrastructurally atrophic, retained little antigen, and produced no iccosomes. By interfering with normal follicular dendritic cell function, age likely has the same effect on transmissible spongiform encephalopathies as has been observed due to dedifferentiation of follicular dendritic cells (*8*). Senescence of the immune system function could interfere with transmissible spongiform encephalopathy pathogenesis in other ways as well, such as impairing migrating intestinal dendritic cells or complement pathways involved in complexing PrP^Res^ to follicular dendritic cells.

This hypothesis could be readily tested by intracerebral versus peripheral PrP^Res^ challenge of young versus old animals. Because the intracerebral challenge bypasses the immune system portal, old, peripherally challenged animals should show a disproportionate reduction in disease risk if immune system senescence is important in regulating pathogenesis.
